# Naturally available wild pollination services have economic value for nature dependent smallholder crop farms in Tanzania

**DOI:** 10.1038/s41598-019-39745-7

**Published:** 2019-03-05

**Authors:** Byela Tibesigwa, Juha Siikamäki, Razack Lokina, Jessica Alvsilver

**Affiliations:** 1 Environment for Development Tanzania (EfDT), Department of Economics, University of Dar Es Salaam, Dar Es Salaam, Tanzania; 2 Environmental Policy Research Unit (EPRU), School of Economics, University of Cape Town, Cape Town, South Africa; 3 International Union for Conservation of Nature (IUCN), 1630 Connecticut Avenue NW, Suite 300, Washington, DC 20009 USA; 4 Swedish Environmental Protection Agency (SEPA), Stockholm, Sweden

## Abstract

Despite the importance of naturally available wild pollination ecosystem services in enhancing sub-Saharan African smallholder farms’ productivity, their values to actual farming systems remain unknown. We develop a nationally representative empirical assessment by integrating nationally representative plot level panel data with spatially and temporally matched land cover maps to identify the contribution of wild pollinators to crop revenue. Our estimation results reveal distinct and robust contributions by natural habitats of wild pollinators - forests - to plot-level crop revenue, where habitats in near proximity to plots contribute much more value than those farther away. When contrasting between *pollinator-dependent* and *pollinator-independent crops*, we find that the positive effects emerge only for *pollinator-dependent* crops, while *pollinator-independent crops* show no benefits. We conclude the empirical assessment by using our estimates to evaluate changes in crop revenue associated with the actual habitat reduction during 2008–2013. We find that this change in the natural habitats of wild pollinators has reduced crop revenue possibly by as much as 29% (mean) and 4% (median). To our knowledge, this is the first empirical assessment to use nationally representative smallholder farms to assess the value of naturally available wild pollination ecosystem services. Our results magnify the documented benefits of forest conservation, as this preserves pollinators’ natural habitats, and by extension its inhabitants, who play an important role in boosting crop yields of nature dependent smallholder farms.

## Introduction

As pollinators continue to take the global spotlight, the value of wild pollinators remains unclear^1–5^. This is particularly concerning for sub-Saharan Africa, because the region is heavily reliant on agriculture, where most farms are smallholder, subsistence^6–8^, and dependent on naturally available ecosystem services for increasing productivity. Anecdotal evidence suggests that these farmers often perceive ecosystem services, such as wild pollination, as an infinite gift from nature^9,10^. This perception, coupled with a lack of ready cash or credit, implies that sub-Saharan African farmers are unlikely to invest in artificial pollination. For these reasons, any decline in wild pollinators, as projected by the current literature^11^, will decrease crop yields and threaten already dwindling household food security, creating further obstacles to achieving the United Nations’ Sustainable Development Goals (SDGs). Here, we measure the value of wild pollinators to smallholder crop production in Tanzania, using rigorous panel data estimation and nationally representative data on actual small-holder farms. Our results reveal the important contribution by forests, as natural habitats of wild pollinators, to crop revenue, and suggest that the value of natural habitats includes their ability to support wild pollinator populations. We also find that forests in close proximity to farm plots contribute much more value than those at a farther distance, strikingly revealing an exponential decline. When we contrast pollinator-dependent and pollinator-independent crop revenue, we find significant positive gains from proximity to forests for the pollinator-dependent crops. Our results suggest that one way of improving crop yield, and meeting the SDGs, is to re-emphasize the preservation of natural forest ecosystems adjacent to current and potential future farmland.

Mirroring most countries in sub-Saharan Africa, Tanzania’s agriculture continues to be the backbone of the country, with 75% of its population residing in rural areas^12,14^. Agriculture is a main source of livelihood, boosting household income and food security; close to 85% of rural and 15% of urban Tanzanians rely on agriculture^12^. Nationally, agriculture accounts for 25–30% of GDP; it also contributes to export earnings and about 73.6% of total employment^12,13^. Therefore, agriculture is a main ingredient in Tanzania’s strategies to achieve sustainable development in response to pervasive, dire poverty^14,15^. Despite polices to transform agriculture into a modern engine of growth^12,13,16^, the sector’s performance is slow, smallholder farms continue to predominate^12,15^, and Tanzania remains a peasant economy^12^. Smallholder farms, prevalent in sub-Saharan Africa, often serve the dual role of supplying food for the household and providing products for markets to generate income^6,7^, intrinsically linking smallholder farming to household food security^7,15^. Because smallholder farms usually lack access to modern methods, they are vulnerable to any changes in natural ecosystem services that support agricultural production. Tanzania is abundantly endowed with such resources, including habitats favourable for pollinators. There are many wild bee colonies in natural habitats, as well as beekeeping activities, maintained to generate food and raw materials for industries^17,18^. However, managed colonies are not rented out to boost agricultural productivity. This is similarly observed in other parts of sub-Saharan Africa, where there is a lack of awareness of the link between pollinators and crop productivity, and a lack of a market for pollination services^19–21^. In such settings, where markets and credit are often lacking, and where there is heavy reliance on nature, it becomes imperative to document the economic value of ecosystem services such as wild pollination. To our knowledge, this is the first study with direct linkages to actual farming systems^3^; past studies have been based on expert assessments or experimental conditions^5,22–26^.

**Figure 1:**
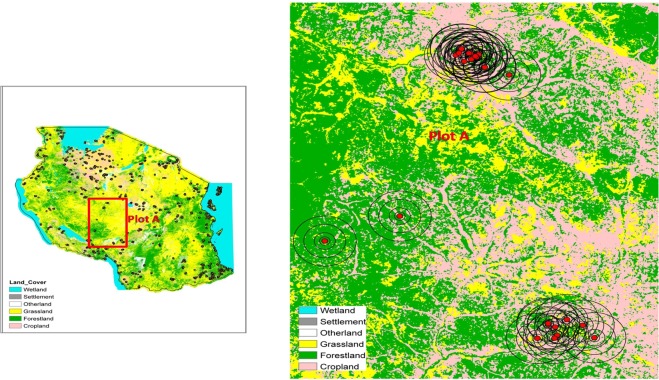
Land cover showing the wild pollination proxy. The left hand side shows the distribution of small-holder farms in Tanzania. The red dot represents the plot location. The right hand side shows the wild pollination proxy where we use distance (and density) between natural habitats and farm plots to measure the availability of wild pollination services around each plot. These are the concentric circles/ buffers (100 m, 250 m, 500 m, 1000 m, 2000 m, and 3000 m radius) around each of the plots in the Tanzania NPS data using the NASA SERVIR land cover maps.

Our estimation uses the National Panel Survey (NPS), a representative survey (see Methods) of households who engage in smallholder agriculture, and, on average, own two plots, which are about two acres per plot. The crop data is available at plot level; Fig. 1 shows the distribution of the farm plots in Tanzania. This is important to our estimation, as we use the geo-coordinates to locally match land cover with the location of the plot. Smallholder farmers grow a variety of crops in a single plot targeting the two rainy seasons (“long rains” and “short rains”) and there are crops that are grown throughout the year. Because crops differ in their responsiveness to pollinators, i.e., the extent of yield improvement, we factor this in as an identification strategy by using the Food and Agriculture Organisation of the United Nations^27,28^ pollination dependency categories: (i) *essential:* crop production is reduced by more than 90% in the absence of animal pollinators; (ii) g*reat:* 40–90%; (iii) *modest:* 10–40%; (iv) *little:* 0–10%; (v) *shows an increase in seed/breeding/yield* in response to pollinators; (vi) *does not show an increase* in yield in response to pollination; (vii) *unknown*: no literature. We group categories (i)-(v) and refer to these as *pollinator-dependent crops*. We call crops that do not depend on pollinators, that is, category (vi), *pollinator-independent crops*. Accordingly, our outcome variables include crop revenue per acre from *all crops*; crop revenue per acre from *pollinator-dependent crops*; and crop revenue per acre from *pollinator-independent crops*. Our wild pollinator measure is proximity to wild pollinators, proxied by the distance from farm plot to forests. The intuition underlying this proxy is that forests are natural habitats of wild pollinators^29–31^, and we hypothesize that there is a relationship between crop productivity and the foraging distance and frequency of pollination, which is based on the availability of nectar and pollen within flight distance^32–34^. In constructing the proxy, we use the SERVIR land cover maps and construct concentric circles (buffers) from the edge of the farm plot (Fig. 1), of radii sizes 100 m, 250 m, 500 m, 1000 m, 2000 m and 3000 m, measuring the amount of forest within each buffer, thereby testing the hypothesis that wild pollination is likely to decrease with increases in distance. The proxy captures wild animal pollinators in general, that is, wild bees and other insects including birds and mammals, as we are unable to distinguish the contribution of each to crop production. However, given that bees are the dominant wild pollinators, it is reasonable to assume that the proxy mostly captures the services offered by wild bees. Our estimation strategy is based on a fixed-effects production function model, where pollination is assumed to be an additional input to production where we include a series of controls. See Methods.

## Results and Discussions

### Smallholder crop diversity and responsiveness to pollinators

There are over 40 different crops grown during the long-rains, that is, Masaki season (February/March to June/July). Out of these, three are non-food crops (tobacco, cotton and sisal), while the rest vary between grains, tubers, fruits and vegetables. The most common long-rains crops are maize (41.9%), paddy rice (12.6%), beans (9.8%), groundnuts (4.7%) and sorghum (4.3%). During the short-rains, that is, Vula season (September/October to January/February), there are slightly less than 40 different crops; these are mainly food crops, except for three crops, that is, cotton, tobacco and rubber. The most common crops in this season include maize (48.4%), beans (17.0%), sweet potatoes (5.7%), paddy (5.4%) and groundnuts (4.1%). There are more than 30 permanent fruit crops; the largest of these are bananas (15.6%), mangoes (14.5%), cashew nuts (8.6%) and coconut (5.0%). Overall, the majority of the crops grown by smallholder farmers appear to be pollinator dependent, but this dependence varies from season to season, further results are available upon request.

### Contribution of wild pollinators - forests, their natural habitats - to crop revenue by distance from the smallholder farm plot

**Figure 2:**
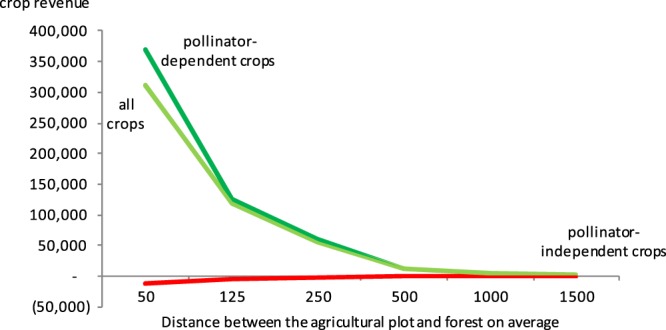
Per acre forests contribution to crop revenue per acre, by distance from plot. Estimates from Table 1 are used to derive value of forest to revenue per acre. The marginal value of forests, per acre, is declining with distance between forest and farm plot. An exponential function predicts well the marginal benefit curve. Note: US$1 ≈ TSH2000.

Table 1 reports the estimation results from fixed-effects models. Only the estimates, standard errors and p-values are presented here; the full regression models are reported in Supplementary Information Tables S2–S4. The regression models were run separately for each buffer, that is, 100 m, 250 m, 500 m, 1000 m, 2000 m and 3000 m buffer. Panel 1 outcome is revenue earned from *pollinator-dependent crops*, while the outcome in Panel 2 is revenue from *all crops*, and Panel 3 uses revenue from *pollinator-independent crops*. The results, in Panels 1 and 2, reveal that forests increase the total revenue earned from crop farming. That is, in Panel 1, we observe a positive relationship between forest share and crop revenue from *pollinator-dependent crops*, and this relationship is significant for all models except for the 100 m buffer model. A somewhat similar pattern is observed in Panel 2, although this time the size of the coefficients are smaller, which is a likely effect of the outcome variable, which is a summation of revenue earned from *all crops*, including *pollinator-independent crops*. Results on *all crops* are particularly relevant as they denote the total impact of the availability of wild pollinator habitats on total crop revenues, considering as such any options the farmer has to adapt to changing wild pollinator availability (for example, by switching to pollinator independent crops if wild pollinators decline). Another noteworthy observation is the fact that the size of the coefficients increases as we move from the 100 m buffer to the next, all the way to the 1000 m buffer, and thereafter we observe a slight decline in the 2000 m and 3000 m buffer. This suggests forest cover yields no additional benefits for forests beyond 2–3 km from the agricultural plot. Note, however, that the estimates denote the effects of forest share in each buffer, and not the contribution of forest per hectare. That is, larger buffers have larger surface areas, hence the evaluation of per acre contributions of forest needs to consider the number of acres in each buffer. For example, a smaller coefficient estimate (11383) within a 100 m buffer does not automatically indicate that the value per acre of forest is smaller than for the1000m buffer, which has larger coefficient (22667). In order to identify the value, we use the estimates in Table 1 to derive the value of forest to total revenue per acre. The results are shown in Fig. 2, which shows the marginal value of forests, per acre, declining with distance between the natural habitants of wild pollinators and farm plot. An exponential function predicts well the marginal benefit curve.

**Table 1:** Estimation results from panel regression models to predict crop revenue from pollinator-dependent crops, all crops and pollinator-independent crops.

	Panel 1 Crop revenue, pollinator-dependent crops			Panel 2 Crop revenue, all crops			Panel 3 Crop revenue, pollinator-independent crops		
	Estimate	Standard error	P-value	Estimate	Standard error	P-value	Estimate	Standard error	P-value
Forest share, 100 m radius	11383.57	6347.59	0.107	7265.588**	2854.496	0.031	−68.53881	426.2044	0.876
Forest share, 250 m radius	14840.926**	6329.108	0.044	9989.2996***	1898.507	0.001	−879.6146	567.4782	0.156
Forest share, 500 m radius	22248.856***	6806.572	0.010	16049.671***	3815.392	0.002	−971.1914	535.2411	0.103
Forest share, 1000 m radius	22667.803**	7696.566	0.016	19347.769***	5045.113	0.004	−325.4498	776.2274	0.685
Forest share, 2000 m radius	20705.675**	8073.948	0.030	17952.209***	3927.534	0.001	−21.38573	931.9754	0.982
Forest share, 3000 m radius	21684.896**	8523.845	0.032	18723.087***	4195.593	0.002	−191.2735	865.5609	0.830
Sample size	10214			10214			10214		

Land cover measured using SERVIR land cover map. The estimation results come from panel regression models estimated separately for each radius 1–6. ***p < 0.01, **p < 0.05, *p < 0.1. The estimation models control for plot characteristics (soil quality; slope; distance to farm road and market), production inputs (expenses in labor, fertilizer, seed), farmer characteristics (age, education, agricultural extension services, female versus male headed households, off-farm employment), and weather (temperature and rain). The full models are shown in Tables S6–S8. Note: US$1 ≈ TSH2000.

### Placebo tests and robustness checks

In general, identification requires that unobservable factors that influence agriculture not be systematically correlated with pollination. Although this assumption may be somewhat untestable, we attempt to do so using placebo regressions (Panel 3), where we take advantage of the fact that small-holder farms grow a variety of crops. That is, unlike Panels 1 and 2, which show revenue per acre from *pollinator-dependent crops* and *all crops* respectively, the placebo regressions consist of revenue per acre of *pollinator-independent crops*. Hence, if indeed identification has been achieved, then the coefficient will not be significant in our placebo test. The test offers satisfactory results, as evident in Panel 3 and Fig. 2. That is, we find insignificant coefficients, suggesting that our previous results in Panels 1 and 2 are unlikely to be picking up any time-varying unobservables. The second test uses different land cover maps – Hansen *et al*. (2013) and Globcover data (http://due.esrin.esa.int) – to determine whether consistent results will be obtained. The main difference between Hansen *et al*. (2013) and SERVIR is that the latter reports a larger share of forest cover in each buffer; upon re-estimation based on Hansen *et al*. (2013) forest cover, we find somewhat larger coefficients and level of significance, but, overall, we obtain qualitative similar results to SERVIR. This is expected, as the fine temporal resolution of the Hansen *et al*. (2013) data enables us to measure land cover more precisely, thus reducing the downward bias associated with the greater measurement error likely associated with our mainland cover data. With the Globcover data, the difference is that it has a 3000 m by 3000 m resolution (instead of 30 m by 30 m), which decreases the likelihood of identifying any relationship. The results are available upon request.

### Change in crop revenue due to change in forests, the natural habitats of wild pollinators

**Figure 3:**
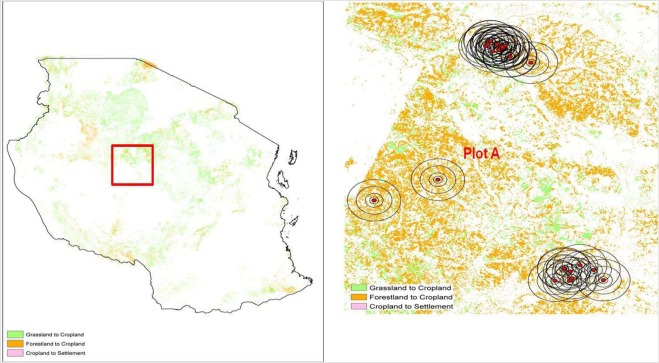
Change in land cover in Tanzania between 2000 and 2013. The figure shows how land cover has been changing in Tanzania under the SERVIR land cover maps between 2000 and 2013. White depicts no change in land cover, brown shows a change in land cover from forest to cropland, and pink shows change in land cover from cropland to settlement.

**Figure 4:**
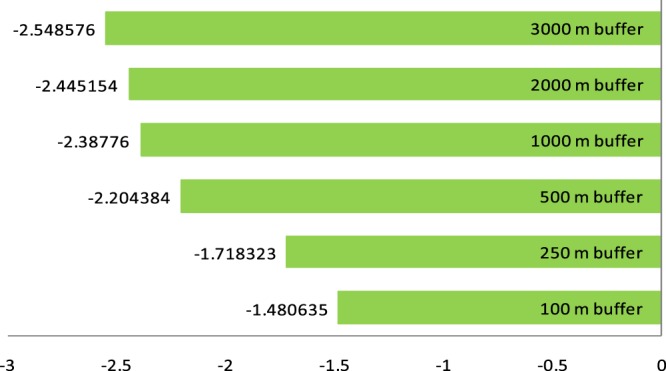
Average % change in the share of forest cover in each buffer. This shows the average percentage change in the forest cover in 100 m, 250 m, 500 m, 1000 m, 2000 m and 3000 m radius buffers (i.e., change in the natural habitats of wild inset pollinators) using the NASA SERVIR land cover maps. Note: US$1 ≈ TSH2000.

**Figure 5:**
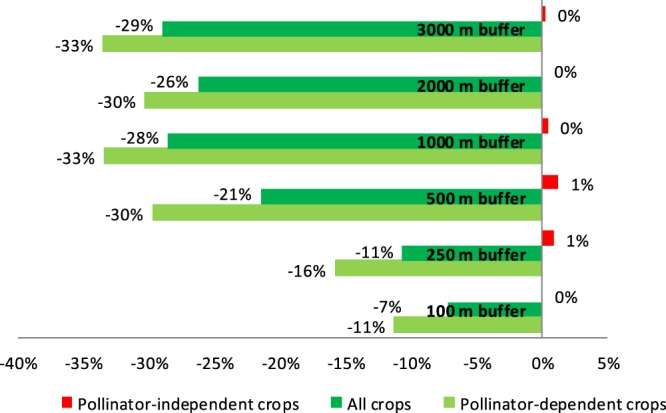
Average % change in crop revenue in each buffer (2008–2013) due to change in the natural habitant (forests). We use the coefficient estimates from Table 1 in combination with the actual change in forest cover between 2008 and 2013, in each plot, in the different buffers, to estimate changes in revenue attributable to forest cover change. From these figures we observe that the decrease in the share of forest cover increases as we move from the 100 m to the 3000 m radius buffer. Here we show the average percentage change in total household crop revenue in 100, 250, 500, 1000, 2000 and 3000 radius buffers. The absolute values for pollinator-dependent crops include TSH-84197.54 (−11% mean), (TSH0, 0% median); TSH-112358.20 (−16% mean), (TSH-1091.87, 0% median); TSH-206398.20 (−30% mean), (TSH10474.70, −2% median); TSH-227545.30 (−33% mean), (TSH18832.80, −3% median); TSH-216671.20 (−30% mean), (18965.50, −3% median ); TSH-228964.20 (−33% mean), (TSH26647.40, −5% median) for the 100 m, 250 m, 500 m 1000 m, 200 m and 3000 m buffer respectively. The absolute values for all crops include: TSH-53739.26 (−7% mean), (TSH0, 0% median); TSH-75627.31 (−11% mean), (TSH734.93, 0% median); TSH-148889.60 (−21% mean), (TSH7556.13, −1% median); TSH-194217.90 (−28% mean), (TSH16074.40, −3% median); TSH-187858.00 (−26% mean), (TSH16443.50, −3% median); TSH-197691.40 (−29% mean), (TSH23007.80, −4% median). Also for the 100 m, 250 m, 500 m 1000 m, 200 m and 3000 m buffer respectively. For the pollinator-independent crops, the values are: TSH506.94 (0% mean), (TSH0, 0% median); TSH6659.41 (1% mean), (TSH64.71, 0% median); TSH9009.55 (1% mean), (TSH457.23, 0% median); TSH3266.95 (0% mean), (TSH270.38, 0% median); TSH223.79 (0% mean), (TSH19.59, 0% median); and TSH2019.60 (0% mean), (TSH235.05, 0% median) for the same buffers respectively. Note: US$1 ≈ TSH2000.

This part of analysis uses the results obtained so far to examine how the recent change in forest cover in Tanzania has affected the crop revenue of smallholder farms. Figure 3 shows land cover change from 2000 to 2013 in Tanzania under SERVIR maps. Most of the change is forest and grassland conversion to agricultural land. At the buffer level, we observe that the reduction in the share of forest cover increases as we move from the 100 m to the 3000 m radius buffer; this decrease in the share of forest varies between 1.48–2.5% (see Fig. 4). To see the responsiveness of smallholder crop revenue to change in pollinators’ natural habitats, we use the estimates in Table 1 in combination with the actual change in forest cover between 2008 and 2013, around each plot, in the different buffers. This is calculated at household level, thus producing household-level crop revenue, with results shown in Fig. 5. We find that the reduction in the share of forests results in the reduction of household-level crop revenue. However, when we include the full effect of pollinator habitat change (accounting for adaptation of crop choice) and use the revenue from *all crops*, instead of *pollinator-dependent crops* only, we find the reduction in household-level crop revenue to be lower.

### Concluding remarks

Determining the value of wild pollination ecosystem services is particularly important for sub-Saharan Africa because it relies heavily on agriculture at the national and household level, and there are high levels of poverty, which means farmers are unlikely to invest in artificial pollination. That is, farming is mainly small-scale and heavily dependent on the natural ecosystem services to boost crop yield. To our knowledge, this is the first empirical assessment of pollination to use nationally representative data on actual smallholder farming systems. Our results demonstrate the benefits of forest conservation to agriculture, further magnifying the overall benefits of forests, which have been quantified in other contexts, such as carbon sequestration^35^. Globally, but more so in sub-Saharan Africa, increasing crop yield often is associated with expanding farms, which implies conversion of the natural ecosystem into farm plots^36–39^, as strongly evident in the land cover change in Tanzania. But this process reduces essential ecosystem services, which decreases crop yields. Given the present importance of agriculture in the region, it is urgent that we identify other ways of increasing crop yields besides expanding farm plots. Our study suggests that a way to improve crop yield is to preserve forests to adjacent farmland, thus enhancing crop productivity per acre. This can increase food security in smallholder farming households and thereby increase the chances of meeting the SDGs in sub-Saharan Africa.

## Methods

### The Production Function Model

In assessing the economic value of pollination services to agriculture productivity, we use the production function approach^40–42^. The production function can be formally presented in Eq (1), where *y* is agriculture output, x is a vector of inputs and costs of production, and q, the pollination service, is considered as an additional input in the production function.

1 $$y={\rm{f}}({\bf{x}},{\rm{q}})$$

Further, p and w are the prices of agricultural output and input respectively. Given the aforementioned, the social welfare, W that is associated with the production function is the area below the demand curve for *y* less input costs:

2 $$W({v}_{I},q)={\int }_{0}^{y}p(u)d-\sum _{i}F{V}_{I}$$

where W is the social welfare, ${\int }^{}({\rm{p}}({\rm{u}}){\rm{du}}$ is the consumer surplus, V_i_ is a vector of agriculture inputs, and F is a vector of agricultural input prices. Under the assumption of price-taking in a perfectly functioning market, the value of the pollination services can be obtained by observing how agricultural output will change in response to change in pollination services, as given by the first order condition:

3 $$\partial W/\partial q=p(\partial y/\partial q)$$

Because pollination service is treated as an additional input, its value is therefore equal to the impact it has on the productivity of agricultural production. The function can be empirically approximated by a panel data model of the form:

4 $${{\rm{y}}}_{{\rm{it}}}={{\rm{\beta }}}_{0}+{{\rm{\beta }}}_{1}{{\rm{q}}}_{{\rm{it}}}+{\bf{x}}{\boldsymbol{\beta }}+{{\rm{\mu }}}_{{\rm{i}}}+{{\rm{\varepsilon }}}_{{\rm{it}}}$$

where the subscripts *i* and *t* represent the *i*-th plot in the *t*-th time period, and, as before, *y* is agricultural output, represented by total crop revenue, of the total quantity harvested, per acre; q is the pollination service, captured by distance to natural resources; x is a vector of inputs, which also includes the plot, household, soil and climate characteristics added as controls; and finally ${{\rm{\mu }}}_{{\rm{i}}}$ and ${{\rm{\varepsilon }}}_{{\rm{it}}}$ are the random disturbances. In this case, the fixed-effects model works well because it accommodates the likely presence of time-invariant unobservables which maybe correlated with our covariates. For example, some district may have invested in more modern and efficient irrigation systems than other districts. However, time-varying unobservables, if present, are likely to produce biased estimates under a fixed-effects model. Because we attempt to include most of the variables that will influence crop yield, this is unlikely to be a limitation in this study.

### Data and Variables Definition

The main data for analysis is the Tanzania National Panel Survey (NPS). The Tanzania NPS is a national representative household survey, which is managed by the Tanzania National Bureau of Statistics (NBS) in collaboration with the Development Research Group at the World Bank. The survey includes four main strata: main city Dar es Salaam, urban areas in the Mainland, rural areas in the Mainland, and the island Zanzibar. Within these strata, multi-stage cluster sampling design is employed, where the clusters in the rural regions are villages, while the clusters in the urban areas are census enumeration areas. The objective of the survey is to monitor poverty dynamics, known as ‘MKUKUTA’ which is a National Strategy for Growth and Reduction of Poverty (NSGRP), and other national policies. As such, the survey covers a broad range of areas: education, labor markets, agriculture; consumption and expenditures, and other development indicators. These development indicators are collected using four main questionnaires: household, agriculture, fishery and community. Agricultural data are available by plot, and, importantly to our study, we have obtained precise geo-coordinates of each plot in the data, enabling us to locally match land cover data with the location of the plot. The survey contains three waves; fieldwork for the first wave occurred in 2008/2009, wave 2 took place in 2010/2011, and wave 3 in 2012/2013. The attrition rate is very low, and noted to be 3% and 4% in wave 2 and 3 respectively.

#### Plot Revenue from crop farming

The analysis uses three outcomes, where we take advantage of the fact that the Tanzanian smallholder farmers grow an array of crops which include fruits, vegetables, tubers, grains, nuts and seeds. Crop production includes an array of plants grown in the long-rains and short-rains and those that are grown permanently in a single plot. Accordingly, the first is total revenue per acre from *all crops*. The second is total revenue per acre of *pollinator-dependent crops*, and the third is total plot revenue per acre of *pollinator-independent crops*. In deriving our outcome variable, we use Food and Agriculture Organisation of the United Nations^27,28^ pollination dependency categories: *Essential:* crop production reduces by more than 90% in absence of animal pollinators (for example, papaw, passions). *Great:* where crop yield reduces by 40–90% (for example, mango, avocado); *Modest:* production is reduced by 10–40%. (for example, sunflower, coffee); *Little:* production decreases by 0–10% (for example, beans, groundnut); *Shows an increase in seed/ breeding/yield* in response to pollination (for example, cassava, cocoyams); *Doesn’t show an increase* in yield in response to animal pollination (for example, maize, paddy); *Unknown***:** No literature (e.g., monkey-bread, sisal grown by farmers in Tanzania). We use these categories to aid in identification and to check the robustness of our results. We group together ‘essential’, ‘great’, ‘modest’, ‘little’ and ‘increase’ and refer to this as *pollinator- dependent crops*. Our second category comprises crops that do not depend on pollination, that is, ‘Doesn’t show an increase’; we call these *pollinator-independent crops*. We eliminate ‘Unknown’ from the analysis because it is unclear how those crops will respond to pollination. Note that plot revenue per acre is the revenue generated from all crops harvested, divided by the size of the plot, where plot size is measured in acres.

#### Wild pollination ecosystem services

We use two alternative land cover data sets to construct the pollination measure. The first is the NASA Servir Land Cover Data. The Land Cover maps have been developed from LandSat Imagery (30 m by 30 m) resolution using supervised classification in 2000, 2010 and 2013. The data set uses Intergovernmental Panel on Climate Change (IPCC) land cover categories for Scheme I and consists of forest, grassland, wetland, cropland, settlement and other lands. The advantage of the NASA Servir Land Cover Data is that it is specifically for Tanzania; the disadvantage is that it does not cover 2008. Hence we use linear interpolation, using moving averages, to fill in missing land cover for 2008. We use density of natural habitats and their distance from the farm plot to measure the availability of wild pollination services in each plot. This approach can be found in a number of studies^43^, including natural experiments^44^. Note that the pollination measure captures wild animal pollinators in general, that is, wild bees and other insects (for example, ants), birds, and mammals (for example, bats). That is, we cannot distinguish the importance or contribution of each one of these wild animal pollinators to agricultural production. However, given that bees continue to be the most dominant wild pollinators, we can assume that our measure mostly captures the services offered by wild bees. In relation to pollination, forests and grasslands are more likely to be natural habitats for wild pollinators, especially forests, in comparison to the other classifications which are more likely to experience pollinator loss^29–31,44^. For example, a detailed description has been provided on the correlation between land cover and bee colony losses^44^. Based on this, we determine the type of land cover found around the farm plot. Because our outcome is crop revenue of the farm plot, we construct concentric circles (buffers), with various radii from the edge of the farm plot.

In constructing the buffers, we considered two important factors. The first is foraging distance. Essentially, forage is the availability of food supply, that is, nectar and pollen, within flight distance for pollinators. The foraging range is therefore very important for pollination and agricultural productivity. The range varies by species (for example, the *Bombus spp*. outperforms the *Apis mellifera*), season, body size and colony density. The maximum foraging distance for *Bombus spp*. ranges between 870 m to 3900 m^32,34,45^, while the maximum distance for *Apis mellifera* is between 1074 m and 1408 m. Other studies estimate the maximum foraging distance as 312–625 m for *Bombus spp*. After a review of literature, we use an average maximum foraging distance of 750m^44^. The second factor is the foraging frequency. This has been found to decline with increases in distance. For instance, it has been observed that most (40%) of the worker bees foraged within 100 m from the colony, while the remainder were found within 200 m^45^. This has been found to be an exponential format. Based on this and as shown in Fig. 1, our radii are of the following sizes: 100 m, 250 m, 500 m, 1000 m, 2000 m, and 3000 m, with the assumption that pollination is likely to decrease with increases in the radius. Within each buffer, we determine the percentage distribution of each type of land cover; see Fig. 1 and Supplementary Information Fig. S1.

#### Production Inputs

Production inputs include costs incurred by providing: Labour, fertilisers and seedlings, in each plot. *Labour* cost is the total wages paid to hired labourers for land preparation, ridging and fertilizing, and weeding and harvesting crops. This is then divided by the plot size. *Fertiliser* cost is the total value of pesticides, herbicides, organic and inorganic fertilisers used in each plot, divided by the plot size. *Seedling* cost is the total amount used to purchase seeds divided by the plot size.

#### Plot and Household Characteristics

*Steep slope* takes a value of one if the slope is steep and zero otherwise. *Soil quality-good*, that is, highly fertile, takes the value of one if the soil is fertile and zero otherwise. *Extension Service-price information* is represented by one if the household receives information and zero otherwise. *Household head education* refers to the number of years of education. *Gender of household head*, is one if the head of household is female and zero otherwise. *Age of household head* is age in years. *Number of livestock* is an aggregation of livestock owned by the household.

#### Weather

For weather characteristics, we use global climate data. Tanzania has both unimodal and bimodal rain seasons. Areas with a unimodal rain pattern receive only the main rainy season, referred to as Masika, while areas with a bimodal rain pattern receive rain in two seasons, one being the short-rains season, known as Vuli, and the other being the long-rains season, that is, Masika^46^. The short-rains season is from September/October to January/February while the long-rains season is from February/March to June/July. The regions which receive the Masaki and Vuli rains include Mara, Arusha, Kilimanjaro, Tanga, Morogoro, Mbeya, Coast, Kagera, Kigoma and Mwanza^47,48^. Because of this, we average the temperature and precipitation data following the short-rains and long-rains seasons in each year. In addition, we follow the current agro-economic literature and allow for a non-linear relationship between weather and crop revenue.

### Descriptive Statistics

Supplementary Information Table S1 shows the descriptive statistics. Concerning revenue, the average total revenue per acre at the plot level is TSH 434,462. On average, more revenue is earned from pollinator-dependent crops (239,233) than from pollinator-independent crops (105,642). Note that US$1 ≈ TSH2000. Most of the plots are located closer to roads than either the household home or closest market, as is evident by the descriptive statistics, which show average distances of 1.64 km, 2.49 km and 9.82 km respectively. Supplementary Information Table S1 shows a high variation in farm costs. The average labour cost for land preparation, weeding and harvesting is TSH 7,592. An additional TSH 7,014 is used to purchase fertilisers, while TSH 2,539 is used to purchase seeds. The average head of household is approximately 50 years old, with approximately 8 years of education. Further, only 22% of heads of households are women, and 12% have off-farm employment. On average, households own 24 livestock, and 20% of them have received extension advice in the form of price information. Approximately 46% of the households reported using plots with good soil quality. With regards to the weather characteristics, the average temperature in the short-rains is about 40.5 °C, and this increases slightly to 42.6 °C during the long-rains. As expected, the average precipitation during the long-rains is higher than that found in the short-rains, that is, 184.4 mm and 140.5 mm respectively. Concerning forest cover, the NASA Servir Tanzania land cover offers a different percentage share of forest cover in each buffer. That is, in the 100 m buffer, it shows 18.7% of forest cover; moving to the 250 m buffer, it is 19.3%, and this proceeds to 3000 m buffer, which shows 21.1%.

## Supplementary information

Supplementary information
