# Supplementary material for: Naturally available wild pollination services have economic value for nature dependent smallholder crop farms in Tanzania
Source: Sci Rep. 2019 Mar 5;9:3434. doi: 10.1038/s41598-019-39745-7 (PMC6401091; doi:10.1038/s41598-019-39745-7)
Supplement: Supplementary file 1 — Supplementary information [file 41598_2019_39745_MOESM1_ESM.pdf]

Naturally available wild pollination services have economic value for nature dependent smallholder crop farms in Tanzania

Supplementary Information

Byela Tibesigwa[1,2], Juha Siikamäki[3], Razack Lokina[1] and Jessica Alvsilver[4]

[1]Environment for Development Tanzania (EfDT), Department of Economics, University of Dar Es Salaam, Tanzania
[2]Environmental Policy Research Unit (EPRU), School of Economics, University of Cape Town
[3]International Union for Conservation of Nature (IUCN), 1630 Connecticut Avenue NW, Suite 300, Washington, DC 20009, USA
[4]United Nations Development Programme (UNDP), Bangkok, Thailand
Corresponding author: byela.tibesigwa@gmail.com

	Variable	Description	Mean	Std. Dev.
	**Outcome**			
•	Total revenue p/a, all crops	Total plot revenue per acre from all crops (TSH)	434461.50	9524036.00
•	Total revenue p/a, pollinator-dependent crops	Total plot revenue per acre from pollinator-dependent crops (TSH)	239232.90	8396685.00
•	Total revenue p/a, pollinator-independent crops	Total plot revenue per acre from pollinator-independent crops (TSH)	105642.10	1379371.00
	**Household & Farm Characteristics**			
•	Distance to home	Distance of plot from home (km)	2.49	11.59
•	Distance to road	Distance of plot from road (km)	1.64	3.27
•	Distance to market	Distance of plot from home (km)	9.82	12.93
•	Labor cost	Cost of hire labour (TSH)	7592.01	40013.93
•	Fertiliser cost	Cost of organic and inorganic fertiliser (TSH)	7014.13	59149.23
•	Seed cost	Cost of seeds (TSH)	2538.84	23373.05
•	Household head- age	Age of household head (years)	49.70	15.59
•	Household head- education	Education of household head (years)	8.81	2.61
•	Household head- female	Head of household is female (1=yes ; 0=no)	0.22	0.41
•	Household head- off-farm labor	Head of household has off-farm employment (1=yes ; 0=no)	0.12	0.33
•	Household no. of livestock	Total number of livestock owned by the household	24.03	40.58
•	Household extension advice	Household receive information on price (1=yes; 0=no)	0.20	0.40
•	Soil quality good	Soil quality of plot (1=yes ; 0=no)	0.46	0.50
•	Temperature, short-rains	Average temperature during the short-rain season	40.47	9.68
•	Temperature, long-rains	Average temperature during the long-rain season	42.62	10.49
•	Precipitation, short-rains	Average precipitation during the short-rain season	140.47	53.83
•	Precipitation, long-rains	Average precipitation during the long-rain season	184.37	91.53
	**Wild Pollination Ecosystem Services (Forests, natural habitats)**			
•	Natural habitat 100m radius	NASA Servir Tanzania land cover data: up-to 100 m buffer (% share of forest)	18.74	31.54
•	Natural habitat 250m radius	NASA Servir Tanzania land cover data: up-to 250 m buffer (% share of forest)	19.31	28.75
•	Natural habitat 500m radius	NASA Servir Tanzania land cover data: up-to 500 m buffer (% share of forest)	19.71	26.84
•	Natural habitat 1000m radius	NASA Servir Tanzania land cover data: up-to 1000 m buffer (% share of forest)	20.21	25.32
•	Natural habitat 2000m radius	NASA Servir Tanzania land cover data: up-to 2000 m buffer (% share of forest)	20.79	24.01
•	Natural habitat 3000m radius	NASA Servir Tanzania land cover data: up-to 3000 m buffer (% share of forest)	21.08	23.34

Table S1: Descriptive Statistics. This table reports the descriptive statistics of all the variables used in the analysis, i.e., total revenue per acre, weather, plot and household characteristics. Note: US$1 ≈ TSH2000

Outcome: Revenue from Pollinator-dep. Crops	(1) upto100m	(2) upto250m	(3) upto500m	(4) upto1000m	(5) upto2000m	(6) upto3000m
**Natural habitat share**	**11,384**	**14,841****	**22,249*****	**22,668****	**20,706****	**21,685****
	(6,348)	(6,329)	(6,807)	(7,697)	(8,074)	(8,524)
Distance, home-plot	-158,983**	-159,009**	-159,111**	-159,041**	-158,997**	-158,990**
	(61,818)	(61,738)	(61,717)	(61,690)	(61,708)	(61,695)
Distance, road-plot	72,725*	72,896*	73,091*	73,003*	72,941*	72,953*
	(34,537)	(34,524)	(34,340)	(34,290)	(34,464)	(34,577)
Distance, road-market	-2,585	-2,536	-2,543	-2,510	-2,483	-2,466
	(2,284)	(2,287)	(2,322)	(2,356)	(2,340)	(2,330)
Labor cost	-11.52**	-11.54**	-11.61**	-11.63**	-11.60**	-11.58**
	(3.959)	(3.954)	(3.930)	(3.921)	(3.930)	(3.937)
Fertilizer cost	-10.44**	-10.39**	-10.33**	-10.35**	-10.37**	-10.37**
	(4.019)	(3.998)	(3.970)	(3.966)	(3.977)	(3.980)
Seed cost	-9.793	-9.758	-9.696	-9.731	-9.730	-9.734
	(9.925)	(9.899)	(9.856)	(9.864)	(9.883)	(9.898)
Household head_age	39,253**	38,890**	38,115**	38,365**	39,474**	39,854**
	(13,644)	(14,292)	(14,806)	(14,431)	(14,374)	(14,517)
Household head_age square	-354.2**	-352.0**	-344.5**	-344.4**	-354.8**	-359.1**
	(111.8)	(118.5)	(123.0)	(118.4)	(118.4)	(120.2)
Household head_ education	61,010	60,194	59,502	60,460	60,790	60,947
	(46,751)	(47,184)	(47,212)	(46,882)	(47,090)	(47,263)
Household head _female	-184,482***	-180,341***	-172,836***	-171,869***	-174,652***	-178,153***
	(19,798)	(19,926)	(20,157)	(20,398)	(21,657)	(22,117)
Household head _off-farm labor	-270,028*	-271,976*	-276,459*	-272,886*	-264,421*	-263,982*
	(136,993)	(135,908)	(132,736)	(133,491)	(137,373)	(137,409)
Household no. of livestock	-702.7	-703.2	-719.6	-751.4	-771.2*	-780.1*
	(403.7)	(412.4)	(418.2)	(410.2)	(411.7)	(416.5)
Household extension advice	149,426***	153,623***	150,371***	150,547***	150,119***	150,686***
	(34,366)	(32,684)	(31,947)	(30,812)	(31,588)	(31,921)
Soil quality is good	63,396	63,664	60,003	57,562	58,819	59,328
	(47,014)	(47,357)	(49,081)	(48,915)	(49,430)	(49,210)
Slope is steep	108,223	107,199	109,171	110,319	109,783	110,344
	(294,284)	(295,663)	(295,515)	(294,739)	(294,264)	(294,076)
Temperature, short-rains	-1.677e+06***	-1.650e+06***	-1.596e+06***	-1.590e+06***	-1.653e+06***	-1.663e+06***
	(116,239)	(116,793)	(114,620)	(120,733)	(103,639)	(100,846)
Temperature, short-rains squared	15,494***	15,209***	14,605***	14,554***	15,340***	15,398***
	(2,421)	(2,342)	(2,324)	(2,366)	(2,154)	(2,135)
Temperature, long-rains	4.534e+06***	4.541e+06***	4.548e+06***	4.565e+06***	4.584e+06***	4.609e+06***
	(944,633)	(944,284)	(943,484)	(949,239)	(952,502)	(953,265)
Temperature, long-rains square	-51,547***	-51,584***	-51,622***	-51,791***	-51,999***	-52,240***
	(10,285)	(10,284)	(10,282)	(10,344)	(10,374)	(10,379)
Precipitation, short-rains	43,135***	43,521***	44,193***	44,520***	44,572***	44,674***
	(10,833)	(10,929)	(10,983)	(11,086)	(11,160)	(11,158)
Precipitation, short-rains squared	-95.42***	-96.59***	-98.47***	-99.26***	-99.31***	-99.61***
	(27.32)	(27.60)	(27.78)	(28.00)	(28.19)	(28.21)
Precipitation, long-rains	-874.1	-999.2	-1,159	-1,159	-1,015	-907.1
	(1,974)	(1,928)	(1,922)	(1,970)	(2,021)	(2,054)
Precipitation, long-rains square	-6.071*	-5.814*	-5.481*	-5.417*	-5.761*	-6.031*
	(2.702)	(2.584)	(2.555)	(2.643)	(2.707)	(2.783)
Wave 2	264,746***	265,776***	270,568***	267,101***	261,460***	258,964***
	(75,974)	(74,824)	(74,740)	(73,688)	(73,362)	(73,639)
Wave3	-228,835***	-226,343***	-209,613***	-209,340***	-214,838***	-211,963***
	(33,080)	(35,495)	(34,205)	(35,315)	(35,146)	(35,859)
Constant	-5.752e+07**	-5.839e+07**	-5.995e+07**	-6.057e+07**	-5.981e+07**	-6.014e+07**
	(2.015e+07)	(2.010e+07)	(2.020e+07)	(2.027e+07)	(2.023e+07)	(2.022e+07)
Observations	10,214	10,214	10,214	10,214	10,214	10,214
R-squared	0.047	0.047	0.048	0.048	0.048	0.048
Number of ID	4,500	4,500	4,500	4,500	4,500	4,500

Table S2: Estimation results from panel regression models to predict crop revenue from pollinator-dependent crops (Forest & Non-forest). Natural habitat within 100m, 250m, 500m, 1000m and 3000m radius buffers are depicted in column 1-6 respectively. **N**on-linear relationship is assumed between weather and crop revenue. Land cover measured using NASA Servir Tanzania data**.** Robust standard errors in parentheses, *** p<0.01, ** p<0.05, * p<0.1. Base: no-forest ha share (%) (aggregation of other land, wetland, settlement, cropland & grassland). Despite the low R-squared, the estimates are likely to be precise due to the large sample size. Note: US$1 ≈ TSH2000

Outcome: Revenue from ALL Crops	(1) upto100m	(2) upto250m	(3) upto500m	(4) upto1000m	(5) upto2000m	(6) upto3000m
**Natural habitat share**	**7,273****	**9,975*****	**16,030*****	**19,368*****	**18,004*****	**18,770*****
	(2,872)	(1,891)	(3,796)	(4,994)	(3,855)	(4,143)
Distance, home-plot	-138,917**	-138,942**	-139,027**	-139,016**	-138,983**	-138,975**
	(52,669)	(52,657)	(52,664)	(52,626)	(52,610)	(52,593)
Distance, road-plot	60,162***	60,286***	60,447***	60,448***	60,401***	60,407***
	(15,799)	(15,723)	(15,683)	(15,704)	(15,784)	(15,820)
Distance, road-market	-2,132	-2,099	-2,103	-2,072	-2,048	-2,034
	(2,760)	(2,750)	(2,782)	(2,811)	(2,784)	(2,763)
Labor cost	-6.973	-6.986	-7.038	-7.061	-7.038	-7.023
	(6.988)	(6.988)	(6.996)	(7.003)	(6.994)	(6.995)
Fertilizer cost	-6.652***	-6.621***	-6.579***	-6.579***	-6.596***	-6.594***
	(1.632)	(1.630)	(1.617)	(1.611)	(1.619)	(1.623)
Seed cost	-12.08**	-12.06**	-12.01**	-12.02**	-12.02**	-12.03**
	(5.042)	(5.030)	(5.000)	(4.985)	(5.011)	(5.021)
Household head_age	52,867***	52,606***	52,005***	51,991***	52,930***	53,260***
	(6,587)	(6,363)	(5,948)	(5,882)	(6,103)	(6,140)
Household head_age square	-483.2***	-481.6***	-475.8***	-473.8***	-482.5***	-486.3***
	(72.40)	(67.81)	(60.83)	(60.91)	(65.46)	(66.10)
Household head_ education	106,370*	105,812*	105,259*	105,837*	106,112*	106,251*
	(53,127)	(53,163)	(52,842)	(52,735)	(53,121)	(53,221)
Household head _female	-157,336***	-154,339***	-148,418***	-145,151***	-147,277***	-150,369***
	(28,152)	(26,557)	(25,081)	(23,893)	(24,148)	(24,355)
Household head _off-farm labor	-220,732	-222,019	-225,312	-222,925	-215,569	-215,186
	(227,353)	(226,682)	(225,854)	(225,341)	(225,390)	(224,941)
Household no. of livestock	71.67	70.73	58.06	29.24	11.98	5.159
	(438.4)	(441.8)	(442.7)	(421.0)	(413.9)	(417.4)
Household extension advice	142,660	145,553	143,536	144,190	143,906	144,366
	(135,452)	(134,633)	(133,786)	(132,017)	(133,124)	(133,746)
Soil quality is good	125,844	125,928	123,164	120,248	121,214	121,682
	(84,810)	(84,923)	(85,025)	(83,933)	(84,907)	(85,001)
Slope is steep	399,694*	398,947*	400,234*	401,089*	400,624*	401,106*
	(207,072)	(207,487)	(208,212)	(208,533)	(207,946)	(208,042)
Temperature, short-rains	-1.872e+06**	-1.852e+06**	-1.810e+06**	-1.790e+06**	-1.844e+06**	-1.852e+06**
	(694,111)	(678,879)	(670,499)	(653,087)	(663,722)	(667,570)
Temperature, short-rains squared	19,015*	18,807*	18,331*	18,112*	18,779*	18,828*
	(8,783)	(8,620)	(8,581)	(8,396)	(8,481)	(8,515)
Temperature, long-rains	3.981e+06**	3.985e+06**	3.992e+06**	4.009e+06**	4.026e+06**	4.047e+06**
	(1.645e+06)	(1.647e+06)	(1.649e+06)	(1.650e+06)	(1.651e+06)	(1.655e+06)
Temperature, long-rains square	-45,177**	-45,204**	-45,235**	-45,390**	-45,574**	-45,780**
	(18,894)	(18,916)	(18,923)	(18,931)	(18,932)	(18,980)
Precipitation, short-rains	35,848**	36,130**	36,664**	37,176**	37,253**	37,332**
	(12,149)	(12,237)	(12,382)	(12,452)	(12,412)	(12,418)
Precipitation, short-rains squared	-79.79**	-80.64**	-82.14**	-83.48**	-83.62**	-83.86**
	(29.52)	(29.77)	(30.12)	(30.33)	(30.17)	(30.18)
Precipitation, long-rains	-1,004	-1,084	-1,200	-1,225	-1,104	-1,010
	(1,815)	(1,817)	(1,870)	(1,936)	(1,918)	(1,896)
Precipitation, long-rains square	-5.290*	-5.115*	-4.860*	-4.716*	-5.003*	-5.238**
	(2.340)	(2.328)	(2.241)	(2.281)	(2.237)	(2.233)
Wave 2	178,372	179,294	183,127	181,880	177,117	174,925
	(125,588)	(126,680)	(127,996)	(127,446)	(125,066)	(124,483)
Wave3	-287,390*	-285,249*	-272,409*	-267,776*	-272,074*	-269,697*
	(146,910)	(144,161)	(139,499)	(137,987)	(142,195)	(142,186)
Constant	-4.439e+07	-4.501e+07	-4.623e+07	-4.722e+07	-4.661e+07	-4.689e+07
	(2.929e+07)	(2.952e+07)	(2.997e+07)	(2.997e+07)	(2.973e+07)	(2.977e+07)
Observations	10,204	10,204	10,204	10,204	10,204	10,204
R-squared	0.029	0.029	0.029	0.029	0.029	0.029
Number of ID	4,495	4,495	4,495	4,495	4,495	4,495

Table S3: Estimation results from panel regression models to predict crop revenue from all crops (Forest & Non-forest). Natural habitat within 100m, 250m, 500m, 1000m and 3000m radius buffers are depicted in column 1-6 respectively. Non-linear relationship is assumed between weather and crop revenue. Land cover measured using NASA Servir Tanzania data. Robust standard errors in parentheses. *** p<0.01, ** p<0.05, * p<0.1. Base: no-forest ha share (%) (aggregation of other land, wetland, settlement, cropland & grassland). Despite the low R-squared, the estimates are likely to be precise due to the large sample size. Note: US$1 ≈ TSH2000

Outcome:<br>Revenue from Pollinator-indep. Crops	(1)<br>upto100m	(2)<br>upto250m	(3)<br>upto500m	(4)<br>upto1000m	(5)<br>upto2000m	(6)<br>upto3000m
**Natural habitat share**	**-68.54**	**-879.6**	**-971.2**	**-325.4**	**-21.39**	**-191.3**
	(426.2)	(567.5)	(535.2)	(776.2)	(932.0)	(865.6)
Distance, home-plot	108.5	122.0	122.5	111.2	107.3	109.2
	(85.78)	(87.55)	(85.68)	(78.87)	(76.87)	(77.91)
Distance, road-plot	1,961**	1,938**	1,936**	1,955**	1,962**	1,959**
	(699.7)	(708.4)	(718.6)	(713.3)	(709.3)	(708.7)
Distance, road-market	-404.5	-406.5	-405.7	-405.4	-404.7	-405.5
	(241.7)	(242.7)	(243.4)	(241.2)	(239.0)	(239.2)
Labor cost	1.514**	1.516**	1.519**	1.516**	1.514**	1.515**
	(0.529)	(0.529)	(0.530)	(0.530)	(0.530)	(0.529)
Fertilizer cost	0.302**	0.299**	0.297**	0.301**	0.302**	0.301**
	(0.126)	(0.126)	(0.125)	(0.125)	(0.125)	(0.125)
Seed cost	0.127	0.124	0.122	0.126	0.127	0.127
	(0.276)	(0.276)	(0.277)	(0.278)	(0.279)	(0.278)
Household head_age	2,848	2,890	2,912	2,864	2,846	2,844
	(3,180)	(3,114)	(3,106)	(3,182)	(3,184)	(3,166)
Household head_age square	-20.47	-20.79	-21.03	-20.64	-20.46	-20.44
	(19.12)	(18.60)	(18.50)	(19.26)	(19.21)	(19.00)
Household head_ education	1,284	1,348	1,361	1,294	1,283	1,285
	(2,586)	(2,633)	(2,618)	(2,591)	(2,587)	(2,590)
Household head _female	-23,135***	-23,721***	-23,884***	-23,369**	-23,113**	-23,209**
	(7,063)	(7,120)	(7,212)	(7,657)	(7,756)	(7,539)
Household head _off-farm labor	5,901	5,947	6,133	5,931	5,902	5,844
	(5,535)	(5,321)	(5,270)	(5,476)	(5,350)	(5,350)
Household no. of livestock	287.4	287.7	288.4	288.2	287.5	288.1
	(332.6)	(333.2)	(333.5)	(333.3)	(333.5)	(333.6)
Household extension advice	21,046*	20,729*	20,957*	21,019*	21,052*	21,031*
	(10,361)	(10,265)	(10,317)	(10,402)	(10,431)	(10,421)
Soil quality is good	20,158***	20,300***	20,417***	20,266***	20,147***	20,202***
	(5,316)	(5,227)	(5,187)	(5,075)	(5,053)	(5,086)
Slope is steep	2,147	2,305	2,174	2,132	2,136	2,133
	(7,466)	(7,556)	(7,561)	(7,498)	(7,467)	(7,484)
Temperature, short-rains	-61,828	-65,860	-67,069	-63,465	-61,625	-62,079
	(109,807)	(109,349)	(108,548)	(108,056)	(108,313)	(108,554)
Temperature, short-rains squared	662.8	713.0	725.1	681.5	659.8	665.4
	(1,457)	(1,453)	(1,443)	(1,439)	(1,442)	(1,443)
Temperature, long-rains	9,155	8,182	8,118	8,626	9,160	8,467
	(28,785)	(28,988)	(28,817)	(28,238)	(27,726)	(27,781)
Temperature, long-rains square	-105.6	-100.4	-100.2	-101.6	-105.4	-99.32
	(285.4)	(286.0)	(285.3)	(281.7)	(277.2)	(277.2)
Precipitation, short-rains	387.5	329.9	316.8	362.2	389.3	372.1
	(249.0)	(271.8)	(271.0)	(266.4)	(268.9)	(270.6)
Precipitation, short-rains squared	-1.005	-0.835	-0.800	-0.934	-1.010	-0.963
	(0.801)	(0.865)	(0.853)	(0.806)	(0.793)	(0.805)
Precipitation, long-rains	-37.43	-36.05	-29.27	-34.28	-36.72	-37.45
	(298.2)	(298.5)	(298.6)	(298.5)	(295.0)	(294.5)
Precipitation, long-rains square	0.00522	-0.0120	-0.0220	-0.00449	0.00509	0.00476
	(0.492)	(0.494)	(0.502)	(0.508)	(0.501)	(0.497)
Wave 2	-12,205*	-12,639*	-12,723*	-12,297*	-12,167*	-12,174*
	(5,893)	(6,016)	(5,986)	(5,886)	(5,823)	(5,804)
Wave3	-37,220**	-38,132**	-38,599**	-37,619**	-37,162**	-37,409**
	(12,775)	(12,741)	(12,673)	(12,188)	(12,065)	(12,110)
Constant	1.130e+06	1.257e+06	1.290e+06	1.186e+06	1.126e+06	1.158e+06
	(2.264e+06)	(2.269e+06)	(2.236e+06)	(2.184e+06)	(2.165e+06)	(2.175e+06)
Observations	10,214	10,214	10,214	10,214	10,214	10,214
R-squared	0.037	0.038	0.038	0.037	0.037	0.037
Number of ID	4,500	4,500	4,500	4,500	4,500	4,500

Table S4: Regression Outputs using Aggregated Land Cover (Forest & Non-forest) with pollination independent crops. Natural habitat within 100m, 250m, 500m, 1000m and 3000m radius buffers are depicted in column 1-6 respectively. Non-linear relationship is assumed between weather and crop revenue. Land cover measured using NASA Servir Tanzania data. Robust standard errors in parentheses, *** p<0.01, ** p<0.05, * p<0.1. Base: no-forest ha share (%) (aggregation of other land, wetland, settlement, cropland & grassland). Despite the low R-squared, the estimates are likely to be precise due to the large sample size. Note: US$1 ≈ TSH2000

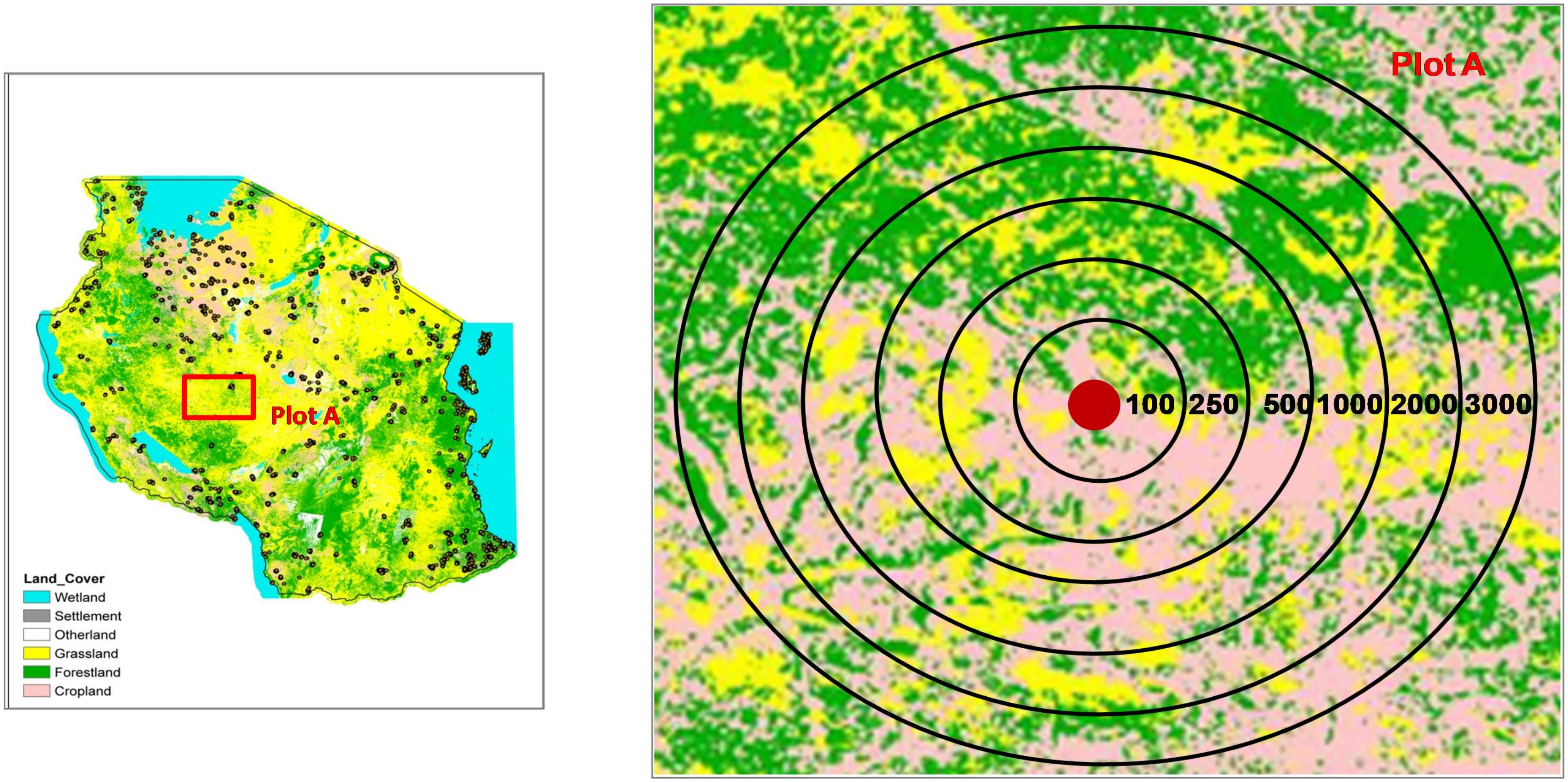

Figure S1: Plot distribution and wild pollination measure/proxy. The left hand side of the figure shows the distribution of the small-holder plot farms, represented by the red dots. The right hand side of the figure shows how the wild pollinator proxy (that is, natural habitats of wild pollinators - forests) was captured. That is, buffers with 100m, 250m, 500m, 1000m and 3000m radius were constructed around each plot, from the edge. We assumed a circular shape of each plot. Using GIS plot information and land cover maps, the type of land cover within each buffer was identified. Thereafter, the percentage share of forest within each buffer is calculated. This is based on SERVIR land cover map.
